# The Impact of Socioeconomic Factors on Pediatric Oral Health: A Review

**DOI:** 10.7759/cureus.53567

**Published:** 2024-02-04

**Authors:** Omar S Almajed, Alhareth A Aljouie, Mayar S Alharbi, Leenah M Alsulaimi

**Affiliations:** 1 Pediatric Dentistry, Imam Abdulrahman Bin Faisal University, Dammam, SAU; 2 Dental Public Health, King’s College London, London, GBR

**Keywords:** preventive dental care, access to oral health care, food security, household income, parental education, life-course socioeconomic disadvantage, oral health-related quality of life (ohrqol), dental caries, socioeconomic status (ses), pediatric oral health

## Abstract

This narrative review examines the impact of socioeconomic status (SES) on pediatric oral health, emphasizing disparities in dental caries prevalence and oral health-related quality of life (OHRQoL) among children from different socioeconomic backgrounds. Utilizing an extensive literature search through PubMed, Google Scholar, and the chat.consensus.app plugin, we synthesized findings from studies published up to December 2023. The review highlights a consistent association between lower SES and adverse pediatric oral health outcomes, influenced by parental education, household income, food security, and neighborhood conditions. It also underscores the importance of the life-course perspective, showing how early-life socioeconomic disadvantages can have long-lasting effects on oral health. Furthermore, the review points to the critical role of school-based oral health education programs and the complex interplay between mental health, SES, and pediatric oral health. By examining the impact of socioeconomic factors across different childhood stages and the effectiveness of educational interventions, this review calls for targeted interventions and policy initiatives aimed at reducing socioeconomic inequalities in pediatric oral health. The findings advocate for a multifaceted approach to improve oral health outcomes for children across socioeconomic backgrounds, ensuring equitable access to oral health care and promoting overall well-being.

## Introduction and background

Pediatric oral health is a critical component of overall child health and well-being, influenced by a complex interplay of various factors, including socioeconomic status (SES). The relationship between SES and pediatric oral health has been the subject of extensive research, revealing significant disparities in oral health outcomes among children from different socioeconomic backgrounds.

Socioeconomic status, encompassing factors like income, education, and employment, plays a pivotal role in determining access to healthcare, dietary habits, and health-related knowledge, all of which are crucial for maintaining oral health. Studies have demonstrated a direct correlation between socioeconomic status and the prevalence of dental caries in children, indicating that children from lower SES backgrounds often experience higher rates of dental caries [1-2]. This relationship is further complicated by factors such as food security, with findings showing that children from food-insecure households exhibited a higher prevalence of untreated dental caries [3].

The impact of socioeconomic factors extends beyond physical health outcomes to affect the oral health-related quality of life (OHRQoL). Research has shown that children from lower SES backgrounds report poorer OHRQoL, suggesting that socioeconomic disparities have far-reaching implications on both the physical and psychological aspects of oral health [4-5]. Furthermore, the influence of parental characteristics, such as education and habits, on children's oral health cannot be overlooked. Significant roles of maternal education and family socioeconomic status in shaping children's oral health behaviors and outcomes have been emphasized in studies [6].

Neighborhood and community factors also play a crucial role. Research has demonstrated that living in a deprived neighborhood is strongly associated with higher caries prevalence among children [7]. This underscores the importance of considering broader socioeconomic and environmental factors in addressing pediatric oral health issues.

Lastly, the life-course perspective provides valuable insights into how early-life socioeconomic factors can have long-term effects on oral health, highlighting the need for early interventions to mitigate these impacts [8].

In summary, the existing body of research clearly indicates that socioeconomic factors are key determinants of pediatric oral health, influencing not only the prevalence of dental diseases but also the overall quality of life related to oral health. This review aims to synthesize these findings to provide a comprehensive understanding of how socioeconomic factors impact pediatric oral health and to identify potential areas for intervention and policy development.

## Review

Methodology

Our study aimed to explore the impact of socioeconomic factors on pediatric oral health through an extensive literature review. We utilized databases like PubMed, Google Scholar, and the chat.consensus.app plugin, employing search terms such as "pediatric oral health", "socioeconomic status", "dental caries", and others related to socioeconomic influences on oral health in children. The search was confined to literature published up to December 2023. It is pertinent to acknowledge that the studies included in this review originate from a diverse range of countries, each with its unique healthcare system and policies regarding oral health care for children, from fully inclusive health insurance models to systems where such coverage is limited or non-existent. This diversity underscores the importance of considering the variability in healthcare systems when interpreting the impact of socioeconomic factors on pediatric oral health outcomes.

For a focused and relevant selection of studies, we established specific inclusion and exclusion criteria, which are detailed in Table 1. The inclusion criteria were peer-reviewed studies that focused on children aged 0-18 years, examining the impact of socioeconomic factors on oral health, utilizing various SES scales, and incorporating diverse study designs. Conversely, the exclusion criteria ruled out studies not directly related to the intersection of socioeconomic factors and pediatric oral health, those published in non-peer-reviewed sources, research focusing on adults, and non-empirical works like theoretical papers or opinion pieces.

**Table 1:** Inclusion and exclusion criteria for the review of socioeconomic factors and pediatric oral health

Criteria	Inclusion	Exclusion
Publication Type	Peer-reviewed studies published in recognized academic journals.	Studies not published in peer-reviewed journals; publications in non-peer-reviewed sources.
Target Population	Studies focusing on pediatric populations (age 0-18 years).	Research focusing on adult populations or those lacking a clear pediatric focus.
Research Focus	Studies examining the impact of socioeconomic factors (income, education, employment, neighborhood environment, food security) on oral health outcomes.	Studies not focused on the specific topic of socioeconomic factors and pediatric oral health.
Study Design	Various study designs including cross-sectional, longitudinal, observational, and interventional.	Theoretical papers, commentaries, or opinion pieces without empirical data.

Data extraction and synthesis involved a narrative approach, where we grouped studies based on the socioeconomic factors examined and their findings. This synthesis aimed to connect different socioeconomic elements with pediatric oral health outcomes, offering a comprehensive view of the current research landscape.

Review

Impact of Socioeconomic Factors on Pediatric Oral Health Across Different Childhood Stages

The impact of socioeconomic factors on oral health varies significantly across different stages of childhood, influenced by a myriad of factors, including access to dental care, dietary habits, parental education, and overall family socioeconomic status. In early childhood (ages 0-5), the prevalence of early childhood caries (ECC) is notably higher in socially disadvantaged populations, with factors such as improper feeding practices, lack of parental education, low familial socioeconomic background, and lack of access to dental care contributing to this trend. The prevalence of ECC can reach up to 85% in these groups [9]. Additionally, socioeconomic status and clinical conditions, including caries and dental trauma, alongside maternal education levels, significantly impact the Oral Health-Related Quality of Life (OHRQoL) in preschool children [10].

As children transition into middle childhood and adolescence (ages 6-15), the association between lower socioeconomic status and higher prevalence of dental caries becomes more pronounced. Factors such as low maternal education, decreased frequency of tooth brushing, and poor oral habits are identified as key contributors to this increased risk [11]. Adolescents from lower socioeconomic backgrounds and poor school contexts exhibit higher levels of dental caries over time, highlighting the persistent influence of socioeconomic inequalities on oral health outcomes [12].

In adolescence (ages 16-18), socioeconomic and clinical factors continue to play a crucial role in shaping oral health outcomes. Caregivers' perceptions of their child's oral health are significantly influenced by clinical and socioeconomic characteristics, including parental habits, dental hygiene, and education level [13]. The evidence underscores the complex interplay between socioeconomic factors and oral health across childhood stages, emphasizing the need for early and targeted interventions to address these disparities and promote equitable access to oral health care [9-13].

The Role of Education and Awareness in Schools in Pediatric Oral Health

Recent studies underscore the significant impact of school-based oral health education programs on improving children's oral health knowledge, attitudes, and practices. For instance, a descriptive cross-sectional survey conducted among school children in Saudi Arabia revealed that only 41.9% of the children had good oral hygiene, highlighting the need for dental health education and intervention within the school curriculum. The study suggests that using a toothbrush and toothpaste effectively for one to two minutes twice a day can significantly improve oral hygiene among children. Furthermore, it emphasizes the importance of parental involvement and the inclusion of oral health care education in school curricula to enhance the dental health of children [14].

Another study that highlights the effectiveness of school-based oral health education is a comparative clinical study testing the effectiveness of school-based oral health education using experiential learning or traditional lecturing in 10-year-old children. This study found that experiential learning programs were more successful than traditional lecturing in improving oral hygiene. Both methods improved the oral health knowledge, attitude, and behavior of children, demonstrating the potential of integrating oral health education into school curricula to foster better oral health practices among students [15].

These findings indicate that educational interventions in schools can play a crucial role in improving pediatric oral health. By integrating oral health education into school curricula, we can foster a more informed approach to oral hygiene practices, dietary habits, and regular dental check-ups, ultimately reducing the prevalence of dental caries and other oral health issues among children in schools

The Interplay Between Mental Health, Socioeconomic Status, and Pediatric Oral Health

The relationship between mental health and oral health in children, particularly in the context of socioeconomic status (SES), reveals a complex interplay of factors that significantly impact children's well-being [1]. Research indicates that children's oral health status is not only influenced by direct clinical conditions but also by broader psychosocial and environmental factors, including family functioning, parental mental health, and SES [16].

A study highlights that children's odds of having good oral health status decrease with increasing parental psychological distress and poor family functioning across all age groups [16]. This relationship is further complicated by socioeconomic factors, which significantly affect children's oral health, particularly among those aged four to seven years, where higher household income is associated with better oral health outcomes [16]. This suggests that interventions aiming to improve child oral health must consider social, economic, and psychosocial dimensions [16].

Moreover, a systematic review and meta-analysis examining the association of oral health status, demographic, and socioeconomic determinants with oral health-related quality of life (OHRQoL) in children underscore the importance of considering the social and environmental contexts in which children live, alongside their oral health status, to effectively enhance children's OHRQoL [17].

Another study explored the impact of SES on OHRQoL among Syrian children with a cleft lip and/or palate, revealing that lower SES levels negatively affect children's OHRQoL [3]. This indicates that children from lower SES backgrounds may require additional psychological and social support to improve their oral health outcomes [18].

Investigations into the influence of maternal common mental disorders (CMD) symptoms on early childhood dental caries found a positive association between maternal CMD and dental caries prevalence in children [19]. This suggests that maternal mental health can significantly impair children's oral health, emphasizing the need for comprehensive health promotion strategies that address both oral health and mental health [19].

These studies collectively highlight the intricate connections between mental health, SES, and oral health in children [16-19]. Addressing these factors through integrated health promotion strategies and targeted interventions can potentially improve both oral health outcomes and the overall quality of life for children across diverse socioeconomic spectrums [16-19].

Impact of Socioeconomic Status on Dental Caries in Children

The relationship between SES and dental caries in children has been extensively studied, revealing significant findings. Research consistently shows that children from lower SES backgrounds tend to have higher rates of dental caries. For instance, a study found that children attending public schools, typically from lower SES backgrounds, had higher overall scores for decayed, missing, or filled teeth and surfaces compared to those in private schools [20]. Parental education, especially maternal education, is significantly associated with a decreased risk of dental caries in children, highlighting the crucial role of parental education in influencing children's oral health [21]. Additionally, food security plays a significant role in pediatric oral health. Children from households with low or very low food security have a higher prevalence of caries as compared to those in fully food-secure households [3]. Family income also influences dental caries, with studies indicating an increased prevalence of caries in children who are underweight and belong to lower socioeconomic status. This underscores the interplay between nutritional status and socioeconomic factors in the development of dental caries [22]. Moreover, living in a deprived neighborhood is strongly associated with early childhood caries, pointing to the importance of community and environmental factors [7]. Maternal oral health is positively linked with the socioeconomic status of the family, affecting caries prevalence in children [23]. A notable study by Dr. Eman Bakhurji et al. in 2020 on Saudi male teenagers found that the use of fluoride toothpaste was associated with lower odds of caries, especially in less advantaged socioeconomic groups, emphasizing the importance of simple oral health practices in mitigating the impact of socioeconomic disparities on dental health [24]. In the study "The Effect of Parental Education and Socioeconomic Status on Dental Caries among Saudi Children" by Passent Ellakany et al. (2021), a clear link was established between higher parental education and income and lower incidence of dental caries in Saudi children. This research, published in the International Journal of Environmental Research and Public Health, emphasizes the significant role of socioeconomic factors in pediatric oral health, particularly in the context of Saudi Arabia, highlighting the need for SES-informed oral health interventions [25].

To provide a clearer understanding of the relationship between socioeconomic status and dental caries in children, Table 2 summarizes key studies in this area. This table highlights the population studied, key findings, and specific SES factors examined in each study.

**Table 2:** Summary of studies on SES and dental caries in children SES: socioeconomic status

Author	Population Studied	Key Findings	SES Factors Examined
Taani et al. [20]	Children in public and private schools	Higher scores for decayed, missing, or filled teeth and surfaces in children from public schools	Type of school attended as a proxy for SES
Tanaka et al. [21]	General child population	Decreased risk of dental caries in children with higher maternal education levels	Parental, especially maternal, education
Chi et al. [3]	Children from different household food security levels	Higher prevalence of caries in children from low or very low food security households	Household food security status
Mangukia et al. [22]	Underweight children from various SES backgrounds	Increased prevalence of caries in underweight children from lower SES	Family income and nutritional status
Willems et al. [7]	Children living in different neighborhood conditions	Strong association between living in deprived neighborhoods and early childhood caries	Neighborhood deprivation level
Shakeel et al. [23]	Families with varying SES	Positive link between maternal oral health and family SES affecting children's caries prevalence	Family SES and maternal oral health
Bakhurji et al. [24]	Saudi male teenagers from different SES backgrounds	Lower odds of caries in less advantaged groups using fluoride toothpaste	SES and use of fluoride toothpaste
Ellakany et al. [25]	Saudi children	Link between higher parental education and income and lower incidence of dental caries	Parental education and family income

Oral Health-Related Quality of Life (OHRQoL) in Children

The OHRQoL in children, influenced by socioeconomic factors, has been a significant focus of recent research. Studies have highlighted various aspects affecting OHRQoL.

A study in Southern Brazil assessed the impact of socioeconomic status and clinical conditions on OHRQoL in preschool children. It was found that older children, those with toothache, and those whose mothers had lower levels of formal education exhibited higher Early Childhood Oral Health Impact Scale (ECOHIS) scores, indicating a negative impact on OHRQoL. Clinical conditions, such as caries and dental trauma, were also associated with poorer OHRQoL [5].

Research in Brazil examining the relationship between socioeconomic backgrounds, clinical factors, and Child Oral Health-Related Quality of Life (COHRQoL) found that untreated dental caries and maxillary overjet were linked to higher impacts on COHRQoL. Children whose mothers had not completed primary education and those from lower-income households reported poorer scores [4].

The severity of early childhood caries and lower family income were found to negatively impact the OHRQoL of preschool children and their parents, emphasizing the role of socioeconomic factors in OHRQoL [10].

A study assessing the relationship between individual and neighborhood social factors and child OHRQoL identified low household income, infrequent neighbor visits, the presence of anterior open bite, dental trauma, and dental caries as determinants of a negative impact on a child’s quality of life [26].

In conclusion, these studies collectively indicate that socioeconomic factors, including parental education, household income, and clinical conditions like dental caries and trauma, significantly influence the oral health-related quality of life in children. This underscores the importance of targeted interventions and policies that address these socioeconomic determinants to improve children's OHRQoL.

To further illustrate the impact of socioeconomic factors on the OHRQoL in children, Table 3 provides a summary of relevant studies. This table offers insights into the populations studied, their key findings, and the SES factors that were examined in relation to OHRQoL.

**Table 3:** Impact of SES on OHRQoL SES: socioeconomic status; OHRQoL: Oral Health-Related Quality of Life; COHRQoL: Child Oral Health-Related Quality of Life

Author	Population Studied	Key Findings	SES Factors Examined
Ortiz et al. [5]	Preschool children	Older children, those with toothache, and children of mothers with lower education had higher ECOHIS scores, indicating worse OHRQoL. Caries and dental trauma also linked to poorer OHRQoL	Age of children, maternal education level, clinical conditions (caries, dental trauma)
Piovesan et al. [4]	Children with different socioeconomic backgrounds	Untreated dental caries and maxillary overjet linked to higher impacts on COHRQoL. Poorer scores in children whose mothers had less education and from lower-income households	Mother's education level, household income, clinical factors (untreated dental caries, maxillary overjet)
Abanto et al. [10]	Preschool children	Severity of early childhood caries and lower family income negatively impacted OHRQoL of children and their parents	Family income, severity of dental caries
Guedes et al. [26]	Children with various social backgrounds	Low household income, infrequent neighbor visits, anterior open bite, dental trauma, and dental caries were determinants of negative impact on child’s OHRQoL	Household income, neighborhood interactions, clinical conditions (anterior open bite, dental trauma, dental caries)

Socioeconomic Influences on Oral Health-Related Quality of Life in Children with Special Needs

In children with cerebral palsy, oral diseases and disorders significantly impacted OHRQoL [27]. This study revealed that dental caries experience and bruxism negatively affected OHRQoL while higher family income was associated with a positive impact. Extending beyond cerebral palsy, the quality of life related to oral health for children with special needs is significantly influenced by a variety of factors, including the severity of untreated dental caries, SES, and specific healthcare needs.

Research has shown that untreated dental caries can have a profound negative impact on the OHRQoL in children with special healthcare needs, affecting both children and their families [28]. This underscores the importance of addressing dental caries as part of comprehensive care for these children.

Socioeconomic factors play a crucial role in the OHRQoL of children with special needs. Studies indicate that lower SES is associated with worse OHRQoL among children with conditions such as a cleft lip and/or palate [18]. This suggests that interventions aimed at improving oral health outcomes in this population must consider the broader socioeconomic context.

Furthermore, the presence of special health care needs itself can influence OHRQoL. For instance, children with a cleft lip and/or palate experience specific challenges that can affect their oral health and overall quality of life, necessitating targeted support and interventions [18]. In addition to these factors, parental perceptions and the home environment also play a role in children's OHRQoL. Parents' educational level, occupation, and oral health literacy can influence how oral health impacts the quality of life of their children with special needs [29].

This highlights the importance of involving parents and caregivers in oral health interventions and education. Overall, addressing the OHRQoL of children with special needs requires a multifaceted approach that considers clinical, psychosocial, and socioeconomic factors. By understanding and addressing these factors, healthcare providers can better support the oral health and overall well-being of children with special needs.

Neighborhood and Community Factors in Pediatric Oral Health

Neighborhood and community factors play a crucial role in pediatric oral health. Research has shown that both the physical environment and social dynamics of a neighborhood can significantly impact children's oral health outcomes.

A study by Guedes et al. (2014) assessed the relationship between neighborhood and individual social networks and COHRQoL. It found that low household income, infrequent neighbor visits, and the presence of dental issues like anterior open bite, dental trauma, and caries were individual determinants of negative impact on a child’s quality of life [26].

Another study by Crouch et al. (2022) examined the association between community-level support and oral health metrics among children. Children living in supportive neighborhoods had a higher likelihood of receiving preventive dental care and were less likely to have tooth decay [30].

Reynolds et al. (2015) explored the relationship between social capital - both family and neighborhood - and the parent-reported oral health of children in Iowa. The study found significant positive associations between child oral health status and neighborhood social capital [31].

These studies collectively highlight the importance of neighborhood and community contexts in shaping pediatric oral health. Factors such as neighborhood safety, social support, and community resources not only influence access to dental care but also affect overall oral health outcomes in children. This underscores the need for community-focused strategies and interventions to improve pediatric oral health, especially in underserved or disadvantaged neighborhoods.

To underscore the role of neighborhood and community factors in shaping pediatric oral health, Table 4 presents a summary of key studies focusing on these aspects. This table details the specific community factors examined, the populations studied, and the principal findings of each study.

**Table 4:** Neighborhood and community factors in pediatric oral health COHRQoL: Child Oral Health-Related Quality of Life

Author	Population	Key Findings	Community Factors Examined
Guedes et al. [26]	Varied	Negative COHRQoL impact from low income, dental issues	Household income, social networks
Crouch et al. [30]	Children in different neighborhoods	Better oral health in supportive neighborhoods	Community-level supports
Reynolds et al. [31]	Iowa children	Positive association between oral health and neighborhood social capital	Neighborhood social capital

Food Security

Food security significantly impacts pediatric oral health, as evidenced by a study conducted by Chi et al. (2014) [3]. This research analyzed data from the National Health and Nutrition Examination Survey, focusing on children aged 5 to 17 years. The study aimed to explore the relationship between household food security and the prevalence of untreated dental caries while also examining the potential mediation of this relationship by socioeconomic status. The findings revealed that about 20.1% of children had untreated caries. Notably, while 62% of households experienced full food security, a significant portion faced varying levels of food insecurity, with 13% at marginal, 17% at low, and 8% at very low food security levels. The study found a strong association between higher socioeconomic status and lower caries prevalence. More importantly, children from households with low or very low food security exhibited a significantly higher prevalence of dental caries compared to those from fully food-secure households. These results underscore the importance of interventions and policies aimed at ensuring food security as a means to combat the pediatric caries epidemic, particularly in lower socioeconomic communities. This study highlights the crucial link between access to adequate food and children's oral health, suggesting that addressing food security could be a key strategy for improving pediatric oral health outcomes.

Life-Course Socioeconomic Disadvantage

The impact of life-course socioeconomic disadvantage on pediatric oral health is a significant area of concern, as evidenced by various research studies. A study published in the "Journal of Public Health" in 2018 by Ramsay et al. found that socioeconomic disadvantage in childhood, middle-age, and older age were associated with complete tooth loss in later life. This study highlighted that socioeconomic disadvantage in middle age had a particularly strong influence on tooth loss in older age, and poor self-rated oral health in older age was influenced by socioeconomic disadvantage across the life course. The study tested different life-course models, including sensitive period, accumulation of risk, and social mobility, and found that the sensitive period for socioeconomic disadvantage in middle age provided the best model fit for tooth loss while the accumulation of risk model was the strongest for poor self-rated oral health [8].

Another study, "Socioeconomic inequalities in oral health in childhood and adulthood in a birth cohort," by Thomson et al., published in "Community Dentistry and Oral Epidemiology" in 2004, demonstrated that adult oral health is predicted not only by childhood socioeconomic advantage or disadvantage but also by oral health in childhood. The study found that changes in socioeconomic advantage or disadvantage are associated with differing levels of oral health in adulthood. This life-course approach appears to be a useful paradigm for understanding oral health disparities [32].

Furthermore, a study titled "Estimating the Effect of Childhood Socioeconomic Disadvantage on Oral Cancer in India Using Marginal Structural Models" by Rao et al., published in "Epidemiology" in 2015, investigated the direct effect of early life socioeconomic conditions on oral cancer occurrence in adult life. The study found that early-life low socioeconomic conditions had a controlled direct effect on oral cancer when smoking, chewing tobacco, and alcohol were separately adjusted in marginal structural models [33].

These studies collectively suggest that life-course socioeconomic disadvantage has a profound impact on oral health outcomes, including tooth loss, oral cancer risk, and self-rated oral health. Addressing socioeconomic factors at various life stages, particularly in middle and older ages, is crucial for better oral health outcomes.

Table 5 provides a concise overview of studies examining the impact of life-course socioeconomic disadvantage on oral health. This table is particularly relevant to our discussion on how early-life socioeconomic conditions can have enduring effects on oral health outcomes, listing the key studies, their findings, and the specific life-course SES factors they investigated.

**Table 5:** Life-course socioeconomic disadvantage and oral health SES: socioeconomic status

Author(s) or Study Name	Population	Key Findings	Life-Course SES Factors
Ramsay et al. [8]	Multi-age groups	Tooth loss linked to SES disadvantage at various life stages	SES in childhood, middle age, older age
Thomson et al. [32]	Birth cohort	Adult oral health influenced by childhood SES and oral health	Childhood SES, adult oral health
Rao et al. [33]	Indian adults	Early-life low SES linked to higher oral cancer risk	Early-life SES, oral cancer risk

Limitations

We acknowledge limitations in our review such as publication bias and the diversity in study designs and socioeconomic factors, which might affect the ability to draw direct comparisons or definitive conclusions. Despite these limitations, our review seeks to provide a broad understanding of the topic, emphasizing significant trends and findings in the existing literature.

## Conclusions

This review highlights the significant influence of socioeconomic status on children's oral health. Key findings demonstrate that lower socioeconomic backgrounds correlate with higher rates of dental caries and poorer oral health-related quality of life in children. These disparities are shaped by various factors, including parental education, household income, food security, and neighborhood conditions. The review also emphasizes the importance of the life-course perspective, showing that early-life socioeconomic disadvantages can have long-lasting effects on oral health. This underscores the need for early and continuous interventions targeting both individual and community-level factors.

In summary, the review underscores the necessity of a multifaceted approach to addressing pediatric oral health disparities. Effective strategies should involve targeted interventions and policy initiatives aimed at reducing socioeconomic inequalities. By focusing on these underlying factors, we can improve oral health outcomes for children across different socioeconomic backgrounds, ensuring equitable access to oral health care and promoting overall well-being.
